# A test battery for evaluation of muscle strength, balance and functional performance in subjects with chronic ankle instability: a cross-sectional study

**DOI:** 10.1186/s13102-023-00669-5

**Published:** 2023-04-13

**Authors:** Sofia Ryman Augustsson, Erik Sjöstedt

**Affiliations:** 1 Department of Sport Science, Faculty of Social Sciences, Linnaeus University, Kalmar, SE-391 82 Sweden; 2 Samrehab, Västervik, Sweden

**Keywords:** Ankle muscle strength, Single-leg stance test, Single-leg hop for distance, Side hop test

## Abstract

**Background:**

Studies investigating ankle condition in subjects with chronic ankle instability (CAI) using an on-the-field test battery are scarce. Understanding which tests that are most challenging for these subjects has the potential to set realistic goals in rehabilitation and return-to-sports criteria. Thus, the primary aim of this study was to investigate CAI subjects regarding strength, balance and functional performance with an easily used test battery that required minimal equipment.

**Methods:**

This study was conducted with a cross-sectional design. A total of 20 CAI subjects, engaged in sports, and 15 healthy subjects serving as a control group, were tested for assessment of strength, balance and functional performance. A test battery was developed accordingly; isometric strength in inversion and eversion, the single leg stance test (SLS), single leg hop for distance (SLHD) and side hop test. The limb symmetry index was calculated to determine whether a side-to-side lower limb difference could be classified as normal or abnormal. The sensitivity of the test battery was also calculated.

**Results:**

The subjects were 20% weaker on the injured side compared with the non-injured side in eversion (p < 0.01) and 16% weaker in inversion (p < 0.01) (Table 2). For the SLS test, the mean score of the injured side was 8 points (67%) higher (more foot lifts) compared to the non-injured side (p < 0.01). The mean distance of the SLHD was 10 cm (9%) shorter for the injured side compare to the non-injured side (p = 0.03). The mean number of side hop was 11 repetitions (29%) fewer for the injured side compare to the non-injured side (p < 0.01). Six of the 20 subjects obtained abnormal LSI values in all five tests whereas none obtained normal values in all tests. The sensitivity of the test battery was 100%.

**Conclusion:**

Deficits in muscle strength, balance and functional performance appear to be present in CAI subjects with the largest impairments in balance and side hop performance, which stresses the need for return to sport criteria for this group of subjects.

**Trial registration:**

Registered retrospectively on 24/01/2023. NCT05732168.

## Background

In sports, the ankle has been shown to be one of the most common injured body site after the knee [1, 2]. The incidence of ankle injury is high in court games and team sports, such as soccer, volleyball, handball and basketball [2–6]. Lateral ankle sprain is one of the most common traumatic musculoskeletal injuries and up to 40% develop chronic ankle instability after this injury [7]. Chronic ankle instability (CAI) is a name for the chronic symptoms that can be developed after a lateral ankle sprain. Typically, severe sprains are followed by additional sprains and a feeling of instability [8]. Several functions can be affected by ankle instability such as range of motion, muscle strength and functional performance [7]. The range of motion is often reduced, perhaps mainly in the acute phase, but it can also be a long-term problem [7]. Both initial and a long-termed reduced muscle strength [7] has been noted, and balance and postural control may be impaired, as a result of ankle sprains [7, 9]. Overall, this often affects the gait, running and the ability to land when jumping and if not managed appropriately, a cascade of negative alterations to both the joint structure and a person’s movement patterns continue to stress the injured ligaments [3].

Common tests to identify possible impaired functions are; balance test, strength test and various forms of jump test [10]. In one study by Park et al. [10], it was noted that the affected side had clearly reduced static and dynamic balance in addition to reduced strength in inversion. However, strength testing was performed isokinetically, using laboratory equipment, which is not usually provided at a clinic. Further, associations were found between jump performance and strength [10]. Yet, no comparisons or correlations were made between hop and balance or between the various functional tests. Sekir et al. [11] investigated the reliability of a test battery, which included test of functional performance, proprioception and muscle strength, in recreational athletes with CAI. It was noted that the various tests provide clinicians with reliable methods for assessing individuals with recurrent ankle instability. However, only the affected ankle of the patient was tested and strength was measured isokinetically. Thus, there appears to be a need for research investigating whether it is possible to detect impairments in CAI subjects using easy-to-use and less expensive assessment tools. Such a test battery could easily be used in clinic and as an on-the-field screening tool for monitoring CAI subjects in sport-based testing environment. On-the-field screening tests could be used in larger groups of people compared to a laboratory setting. Thus, field tests may improve the potential to identify function deficiency and target training goals of athletes with increased risk of re-injury. In addition, despite the fact that several kinds of tests have been described in the literature, and used in clinics, for the evaluation ankle condition in CAI, the literature is not clear regarding which tests that are most challenging for these subjects.

Hence, the primary aim of this study was to investigate subjects with chronic ankle instability regarding strength, balance and functional performance with an easily used test battery that required minimal equipment. A second aim was to evaluate which of these tests that has the highest ability to discriminate impaired function between injured and non-injured ankle in CAI subjects.

## Materials and methods

### Study design and procedure

Data for cross-sectional study, adhering to the STROBE statement [12] were collected during 2021 to 2022 where subjects with chronic ankle instability (CAI) were evaluated with tests of strength, balance and functional performance. Recruitment took place through a local district rehabilitation clinic, where subjects who applied or came via a referral from a doctor for ankle instability problems were introduced to the study and signed up for test session. Contacts were also made with local sports associations and coaches were introduced to the study through email. Once a potential subject has been identified and notified of interest, he or she was contacted by telephone for an in-depth history of injury history and current status. Five tests were used to assess balance, strength and functional performance and a trained physiotherapist collected all data. The tests were performed at the rehabilitation clinic for the CAI subjects and the Sports Science Laboratory at Radix, Linnaeus University, for the healthy subjects. The test session began with a warm up, which consisted of five minutes of ergometer cycling at 100 W followed by 20 repetitions of heel raises on both feet. Height and weight were noted for each subject.

### Participants

Inclusion criteria for the study were men and women with recurrent unilateral ankle problems due to previous sprains, aged 15–40 years, sought treatment on at least one occasion for ankle injury and have had a minimum of three recurrent sprains in the past year. The exclusion criteria were (1) previous ankle fracture with internal fixation surgery, (2) disease / illness that could have effect on the balance or strength (3) newly sprained/injured in the current ankle within a period of eight weeks prior to test session (4) bilateral ankle and/or foot discomfort. A total of 27 CAI subjects were invited to participate. Four subjects were excluded due to newly injured ankle, two due to bilateral ankle instability and one due to previous problems. Finally, 20 CAI subjects were included in the analysis. The subjects were involved in floorball (n = 8), running (n = 5), football (n = 5), equestrian sports (n = 1) and gymnastics (n = 1). In addition, a convenience sample of 15 healthy aged-matched subjects were also recruited in order to establish the specificity and accuracy of the test battery. No significant differences were noted in characteristics (age, height and weight) between CAI and healthy subjects for the overall group comparison (p ≥ 0.15) or between CAI and healthy men (p ≥ 0.16) or CAI and healthy women (p ≥ 0.09) (Table 1).

**Table 1:** CAI and healthy subjects’ characteristics (n = 35)

	CAI women (n = 11)	Healthy women (n = 5)	CAI men (n = 9)	Healthy men (n = 10)	Total CAI sample (n = 20)	Total healthy sample (n = 15)
Characteristics	Mean (SD)	Mean (SD)	Mean (SD)	Mean (SD)	Mean (SD)	Mean (SD)
Age (years)	26 (8)	27 (7)	27 (5)	24 (3)	27 (7)	25 (5)
Height (cm)	167 (7)	164 (4)	181 (6)	182 (7)	174 (9)	177 (10)
Weight (kg)	65 (6)	62 (8)	83 (10)	80 (10)	74 (12)	75 (12)

#### Outcome measures

The primary outcome was ankle function, in subjects with CAIs, which was assessed by a test battery consisting of two muscle strength tests, one balance test and two functional performance tests. Subjects were instructed to wear shorts and athletic shoes accept for the SLS, which was performed barefoot. All subjects performed the tests in the order that they are described below, starting on the non-injured side then continuing on the injured side. The tests are described elsewhere, so a summary including reference is provided for each test below.

#### Muscle strength

Ankle inversion and eversion isometric muscle strength were assessed using hand-held dynamometry, MicroFET2 (Hoggan Health Industries, Inc., Draper, UT), according to previous described protocol [13]. The subjects were examined in a lying in a prone position with the feet outside the edge with one test leader stabilizing the subject’s lower leg. The dynamometer was placed against the lateral edge of the foot, distal to the base of the 5th metatarsal head, to measure eversion and to the medial edge of the foot, near the base of the 1st metatarsal head to measure inversion. The test leader held the dynamometer stationary with one hand while the participants actively exerted a maximal force [13]. Before the test, the subjects tried the directions against the test leader’s hand. Three maximal isometric contractions, measured in Newton (N) with 15 s of rest between each contraction, were measured and the best attempt was used for further analysis. Five minutes of rest was used when changing test direction.

#### The single leg stance test (SLS)

Balance was measured with the Single-leg stance test (SLS) as previous described [14]. The test began with the subject standing on one leg, with his arms straight down, and the other leg held against the calf of the standing leg. When standing steadily, the subject was asked to close their eyes and maintain balance without using opposite legs or arms. The number of times the subject corrected his balance, during 30 s, was documented as *part foot lifts*. Part foot lift was defined as all parts of the foot that left the surface, e.g. toes, outside foot, heel. Putting down the opposite leg also counted as *part foot lift*. The total number of part foot lifts constituted a score that was used as a result.

#### Functional performance

Functional performance was assessed by the Single-leg hop for distance (SLHD) and the 30-sec timed side hop test as previous described [15, 16]. Before each test, the subject had to perform three submaximal test jumps.

For the SLHD, the subject was standing on one leg with the other leg lifted from the floor. Free leg swing was allowed but the hands was placed behind the back. The subject jumped forward as far as possible, taking off and landing on same foot with a controlled landing. The subject had to maintain balance on landing until the test leader had registered the landing position, approximately (2–3 s). The distance was measured in centimeters from the toe at the push-off to the heel where the subject landed. The best jump of two attempts was used further analysis.

For the side hop test, the subject stood on the test leg, with the other leg lifted from the floor, and hands placed behind the back. Two parallel strips of tape, placed 40 cm apart on the floor was used and the subject jumped from side to side as many times as possible during a period of 30 s. The number of successful jumps performed, without touching the tape, was recorded and used for further analysis.

### Statistical methods

Statistics were calculated using IBM SPSS (IBM SPSS Statistics for Windows, Version 27.0. IBM, Armonk, NY). Descriptive data are presented with mean and standard deviation (SD). Due to the relatively small sample in this study, non-parametric tests were used for comparison analysis. Standardized Z-scores were used and summed in order to calculate the total score for the test battery. Differences in strength, balance and functional performance tests as well as total scores, between injured and non-injured ankle, were analyzed with the Wilcoxon signed ranks test. The Mann-Whitney U test was used to compare test values between CAI (injured side) and healthy subjects and any differences between men and women. The Spearman’s correlation coefficient was used to analyze the association between the different tests.

The limb symmetry index (LSI) was calculated to determine whether a side-to-side lower limb difference could be classified as normal or abnormal. The LSI was defined as the ratio of the score of the injured ankle and the score of the non-injured ankle, expressed in percent (injured/non-injured x 100 = LSI). In the present study, an LSI greater than or equal to 90% was classified as normal [17]. The sensitivity of the individual tests and the test battery was calculated as the number of CAI subjects classified as abnormal/total number of CAI subjects × 100, which expressed the percent probability that the tests would demonstrate abnormal LSI in CAI subjects [17]. The five tests were then combined to produce one test battery, which meant that the subjects should perform more than 90% in the injured ankle compared with the non-injured ankle in all tests in order to be classified as normal.

Specificity (= number of healthy subjects classified as normal/total number of healthy subjects) expresses the percentage probability that the tests would demonstrate a normal LSI in the normal subjects [16]. Accuracy (= number of CAI subjects classified as abnormal + number of healthy subjects classified as normal/total number of CAI and healthy subjects) is defined as the percentage probability that the tests would demonstrate a normal LSI in the normal subjects and an abnormal LSI in the CAI subjects. No differences (p ≤ 0.158) were observed between the right and left legs for healthy subjects; therefore, data were analyzed for the right leg only.

Based on a power of 0.80 (α = 0.05), approximately 18 CAI subjects would be required to detect a 20% difference between injured and non-injured ankle in the balance test score. 20% was considered as a minimal clinically relevant difference. Therefore, this study was planned to recruit a minimum of 25 CAI subjects with regard for potential dropouts.

## Results

Of the 20 CAI subjects, 10 had right ankle and 10 had left ankle instability. The test battery total score was significantly better for the non-injured compared to the injured ankle (p < 0.01). The subjects were 20% (18 N) weaker on the injured side compared with the non-injured side in eversion (p < 0.01) and 16% (14 N) weaker in inversion (p < 0.01) (Table 2). For the SLS test, the mean score of the injured side was 8 points (67%) higher (more foot lifts) compared to the non-injured side (p < 0.01). The mean distance of the SLHD was 10 cm (9%) shorter for the injured side compare to the non-injured side (p = 0.03). The mean number of side hop was 11 repetitions (29%) fewer for the injured side compare to the non-injured side (p < 0.01). Differences were found between men and women, in favor for the male subjects, in the strength tests and the SLHD (p ≥ 0.04) (Table 3). Men also performed better at the side hop test on the non-injured side (p < 0.0) but not on the injured side (p = 0.22). No differences were found between men and women in the SLS test for either side (p ≥ 0.26). Differences were found between injured and healthy subject in SLS (p = 0.01), SLHD (p < 0.01) and the side hop test (p < 0.01) whereas no differences were found for the strength tests (p ≥ 0.25). Result for the group of healthy subjects are shown in Table 4.

**Table 2:** Test results for balance, strength and functional performance for injured and non-injured ankle in CAI subjects. Data are presented with mean (± standard deviation) (N = 20)

	Injured side	Non-injured side
	Mean (SD)	Mean (SD)
Eversion strength (N)	73 (± 20)	91 (± 30) *
Inversion strength (N)	72 (± 33)	86 (± 32) *
SLS	20 (± 12)	12 (± 8) *
SLHD (cm)	96 (± 30)	106 (± 22) *
Side hop (repetitions)	27 (± 12)	38 (± 12) *

SLS = Single leg stance

SLHD = Single leg hop for distance

*=significant difference between injured and non-injured side.

**Table 3:** Test results for balance, strength and functional performance in CAI subjects presented by sex. Data are presented with mean (± standard deviation) (N = 20)

	Men	Women	Men	Women
	Injured side	Injured side	Non-injured side	Non-injured side
	Mean (SD)	Mean (SD)	Mean (SD)	Mean (SD)
Eversion strength (N)	84 (± 20)	64 (± 20) *	110 (± 26)	75 (± 23) *
Inversion strength (N)	93 (± 34)	55 (± 18) *	108 (± 32)	68 (± 32) *
SLS	24 (± 18)	18 (± 7)	11 (± 4)	14 (± 10)
SLHD (cm)	112 (± 32)	84 (± 24) *	116 (± 27)	97 (± 13) *
Side hop (repetitions)	31 (± 15)	24 (± 7)	46 (± 11)	32 (± 8) *

SLS = Single leg stance

SLHD = Single leg hop for distance

*=significant difference between men and women.

**Table 4:** Test results for balance, strength and functional performance in healthy subjects. Data are presented with mean (± standard deviation), (N = 15)

	Right leg
	Mean (SD)
Eversion strength (N)	82 (± 25)
Inversion strength (N)	74 (± 9)
SLS	10 (9)
SLHD (cm)	139 (± 33)
Side hop (repetitions)	43 (± 15)

SLS = Single leg stance

SLHD = Single leg hop for distance

### Correlations

For the injured ankle, a relatively strong correlation was found between strength in eversion and inversion (r_s_=0.73, R^2^ = 0.53, p < 0.01). There were moderate correlations between the strength tests and the side hop test (eversion, r_s_=0.59, R^2^ = 0.35, p < 0.01; inversion r_s_=0.56, R^2^ = 0.31, p < 0.001). There were also a moderate correlations between the strength tests and the SLHD (eversion, r_s_=0.51, R^2^ = 0.26, p = 0.02; inversion, r_s_=0.45, R^2^ = 0.20, p < 0.01). A moderate correlation was noted between the two hop tests (SLHD and side hop), (r_s_=0.49, R^2^ = 0.24, p = 0.02) whereas no correlations were found between the SLS and any of the other parameters (p ≥ 0.48).

### Sensitivity, specificity, accuracy and LSI

Sensitivity ranged from 41 to 100% in the CAI subjects, whereas specificity ranged between 80 and 87% in the healthy subjects (Table 5). Accuracy ranged from 59 to 85% and six of the 20 CAI subjects obtained abnormal LSI values in all five tests whereas none obtained normal values in all tests. Six (30%) subjects obtained abnormal LSI values in four of the five tests, seven (35%) in three tests and one in two tests. Among healthy subjects, 7 of 15 (47%) were classified as abnormal in one of the five tests whereas none were classified as abnormal in more than one test. When the five tests were combined to produce one test battery, a sensitivity of 100% was found.

**Table 5:** Results for sensitivity (%), specificity, accuracy and the number (no) of CAI subjects that were classified as abnormal (i.e. with ≤ 90% performance on the injured side compared with the non-injured side) for the five tests (CAI subjects n = 20, healthy subjects n = 15)

	Eversion strength	Inversion strength	SLS	SLHD	Side hop
Sensitivity (%)	65	60	100	45	85
Specificity (%)	80	87	80	80	87
Accuracy (%)	71	71	83	60	86
Abnormal (no)	13	12	17	9	17

SLS = Single leg stance

SLHD = Single leg hop for distance

## Discussion

The main observation in this study was that impairments in strength, balance and functional performance seem to be quite common in CAI subjects. The total score for the test battery was significantly better for the non-injured compared to the injured ankle (p = 0.01). The sensitivity was high for the SLS (100) and the side hop test (85), and thus, provides a high ability to discriminate impaired performance between injured and non-injured ankle in CAI subjects. When the five tests were combined, to produce one test battery, a sensitivity of 100% was found.

Regarding an acute trauma such as an ankle sprain, reduced strength is often noted in eversion and inversion [7]. Strength impairments have also been noted in chronic ankle instability patients [18, 19]. The CAI subjects in the present study had reduced strength with a 20% difference in eversion and 16% in inversion between injured and non-injured ankle, but strength values did not differ compared to healthy subjects. One plausible explanation for the non-existents difference between CAI and healthy subjects might be the difference in disparity between men and women in the two groups. In the group of CAI subjects, 11 out of 20 were women whereas there were only five women (33%) in the group of healthy subjects. Differences were found between men and women, in favor for the male subjects, in the strength tests and these dissimilarities most likely exist in healthy subjects as well. However, due to the restricted sample size we chose not to analyze this further. The sensitivity of the eversion strength test was 65%, with 13 CAI subjects classified as abnormal whereas the sensitivity of the inversion strength test was 60%. The reduced strength in inversion, noted in the present study, is in accordance with the outcome from an earlier study [18], whereas there was no similarity in decreased strength in eversion. In this study [18], patients with chronic mechanical ankle instability were evaluated with strength measurement. The authors found reduced strength despite long-term functional rehabilitation in this group of patients. In another study [19], participants with ankle instability were divided into two categories, mechanical and functional instability, respectively. The authors found an impaired strength in the group with mechanical instability of plantar flexion and eversion whereas the group functional instability did not exhibit any side differences. In the present study, examination prior to participation was not included. Thus, the number of patients that had objective instability versus functional are unknown, although one does not exclude the other. In summary, reduced strength seems to be present in CAI subjects suggesting that continued strength training after rehabilitation is probably important to reduce the risk of re-injury. In addition, the result from the present study, suggests that isometric testing can detect strength deficits in CAI subjects and thus, should be recommended for clinical use.

In the present study, CAI subjects performed significantly worse at the balance test on the injured side. Contrary to this result, Hiller et al. [14] did not found any differences in balance between injured and non-injured ankle. Instead, those with unilateral injury were found to have disabilities on both sides. However, when comparing controls to individuals with instability a two times higher rate of foot lifts was noted for the injured group [14]. In another study [10], it was noted that CAI patients performed 20% better at the non-injured ankle compared to the injured side in a single leg balance test, which is a minor difference compare to the result found in the present study. However, other test methods were used for evaluation of balance which could have influence the results. The differences of 67% in the SLS and the test sensitivity of 100%, noted in the present study, between injured and non-injured ankle suggests that evaluation of balance is of utterly importance in this group of subjects and that rehabilitation should aim to improve balance ability.

Impairments in functional performance were also noted for the CAI subjects in the present study. There was a 29% difference, with 11 repetitions fewer, in the side hop test on the injured side. Seventeen of the 20 CAI subjects were classified as abnormal giving the test a sensitivity of 85%. Comparable to our data, several studies have provided evidence that functional performance are impaired in patients with chronic ankle instability [10, 20, 21]. Ko et al [20] found impaired performance in the group with a history of ankle instability, relative to the control group. However, no comparison was made between healthy and injured side. In addition, we used a more challenging method for the side hop test in the present study according to Gustafsson et al. [16]. Side hop test are a common evaluation instrument in the ankle context but is usually performed with a 30 cm distance instead of 40 cm. In addition, instead of jumping as many times as possible in 30 s, as in the present study, a more common approach are to jump 10 repetitions as fast as possible. However, jumping for 30 s probably better evaluates muscle endurance and the difficulty to maintain the side cutting movement when fatigue. The mean distance of the SLHD was 10 cm (9%) shorter for the injured side compare to the non-injured side (p = 0.050). The difference is in accordance to a study by Park et al. [10], where also a 10% shorter distance was noted in the non-injured side in people with chronic ankle instability. However, the sensitivity of the SLHD, in the present study, was only 45% and only nine of the 20 CAI subjects were classified as abnormal. Thus, the deficits in SLHD performance could be considered as minor compared to the side hop performance and strength impairments. The lack of influence of SLHD on ankle instability has previously been discussed [21]. One theory provided is that a SLHD primarily loads the foot sagitally, compared to the side hop test which has more impact on lateral structures which are same structures that often are affected in an ankle injury [21]. The present study confirms that a SLHD does not appear to be as challenging as other functional tests such as the side hop test. Thus, the side hop test seem to be more useful when screening for functional impairments in CAI subjects.

Not surprisingly, there was a relatively strong correlation between the two strength test (r_s_=0.73) and a moderate correlation between the two hop tests (SLHD and side hop), (r_s_=0.49). Moderate correlations were also noted between the strength tests and the side hop test (eversion, r_s_=0.59, R^2^ = 0.35 and inversion r_s_=0.56, R^2^ = 0.31) and the SLHD (eversion, r_s_=0.51, R^2^ = 0.26, inversion, r_s_=0.45, R^2^ = 0.20) suggesting that maximum muscle strength have an impact on functional performance in this group of subjects. In one previous study, no correlation between strength test and SLHD could be found [10]. However, the test method used in this study consisted of isokinetic strength tests [10], which is different to the isometric method, used in the present study. The somewhat weaker correlation between eversion strength and SLHD in the present study is rather puzzling. The eversion strength was largely impaired compared to the inversion strength and it would therefore be likely to assume an impact on SLHD performance. However, as stated above, the performance deficit of the SLHD was minor compared to the side hop performance suggesting that SLHD is less demanding for CAI subjects. Impairment in eversion might have more impact on neuromuscular control in ankle frontal plane compared to sagittal plane. Still, we can only speculate regarding the impact of strength on neuromuscular control as no biomechanical data was collected.

Correlations could not be found between the balance test and any of the other parameters in the present study (p ≥ 0.48). This is in contrast to previous study where correlation was found between balance and strength in inversion in CAI patients [10]. However, the absence of a correlation between ankle strength and balance has previous been demonstrated in healthy individuals [22, 23]. One plausible explanation for the lack of significant correlations between balance and strength and functional performance noted in the present study might be the test methods used. Assessment of muscle strength was carried out isometrically which may not provide necessary information regarding the nature of a balance test. One previous study revealed that strength training increased strength in subjects with functional ankle instability but did not improve proprioception [24]. Thus, other factors than muscle strength may be of more importance for balance performance in CAI subjects. However, this does not explain the absence of correlations between the balance test and the tests of functional performance. As stated above, the correlations between the strength tests and the side hop test demonstrates the significance of muscle strength on functional performance in CAI subjects. Yet, these correlations might be due to the actual relationship between muscle strength and power rather than balance and neuromuscular control.

One strength of the present study is the use of reliable and valid methods for assessment of both maximum muscle strength, balance and functional performance. Still, some limitations need to be recognized. First, the results may be affected by the small sample size. For this reason, all data are presented at the group level. In order to investigate whether there is any difference between men and women a larger sample is needed. Second, there was no data collected regarding the rehabilitation protocol about previous ankle sprains. It could be speculated that some CAI subjects dropped out from therapy before fully rehabilitated. Information on rehabilitation protocol and return to sport criteria would have provided a broader understanding of the participants’ function and performance.

## Conclusions

Deficits in muscle strength, balance and functional performance appear to be present in CAI subjects with the largest impairments in balance, side hop performance and eversion strength, which stresses the need for better return to sport criteria for this group of subjects. The findings also indicate that the SLHD test does not seem to be sufficiently challenging when it comes to evaluating functional performance in CAI subjects, whereas the more challenging method for the side hop test used in this study could be recommended. The SLS, inversion and eversion strength tests and the side hop test, had high ability to discriminate performance between the injured and the non-injured ankle in CAI subjects.

## Data Availability

The datasets generated and/or analyzed during the current study are available in the Mendeley Data repository, https://data.mendeley.com/datasets/5f85rkjb62.

## Abbreviations

CAI: Chronic ankle instability
SLS: Single leg stance test
SLHD: Single leg hop for distance

## References

[1] Fong DT, Hong Y, Chan LK, Yung PS, Chan KM (2007). A systematic review on ankle injury and ankle sprain in sports. Sports Med.

[2] Halabchi F, Hassabi M (2020). Acute ankle sprain in athletes: clinical aspects and algorithmic approach. World J Orthop.

[3] Feria-Arias E, Boukhemis K, Kreulen C, Giza E. Foot and Ankle Injuries in Soccer.Am J Orthop (Belle Mead NJ). 2018. 47(10). 10.12788/ajo.2018.0096 30481231

[4] Asai K, Nakase J, Shimozaki K, Toyooka K, Kitaoka K, Tsuchiya H (2020). Incidence of injury in young handball players during national competition: a 6-year survey. J Orthop Sci.

[5] McKay GD, Payne WR, Goldie PA, Oakes BW, Stanley JJ (1996). A comparison of the injuries sustained by female basketball and netball players. Aust J Sci Med Sport.

[6] Augustsson SR, Augustsson J, Thomee R, Svantesson U (2006). Injuries and preventive actions in elite swedish volleyball. Scand J Med Sci Sports.

[7] Miklovic TM, Donovan L, Protzuk OA, Kang MS, Feger MA (2018). Acute lateral ankle sprain to chronic ankle instability: a pathway of dysfunction. Phys Sportsmed.

[8] Doherty C, Bleakley C, Delahunt E, Holden S (2017). Treatment and prevention of acute and recurrent ankle sprain: an overview of systematic reviews with meta-analysis. Br J Sports Med.

[9] Wikstrom EA, Hubbard-Turner T, McKeon PO (2013). Understanding and treating lateral ankle sprains and their consequences: a constraints-based approach. Sports Med.

[10] Park YH, Park SH, Kim SH, Choi GW, Kim HJ (2019). Relationship between isokinetic muscle strength and functional tests in chronic ankle instability. J Foot Ankle Surg.

[11] Sekir U, Yildiz Y, Hazneci B, Ors F, Saka T, Aydin T (2008). Reliability of a functional test battery evaluating functionality, proprioception, and strength in recreational athletes with functional ankle instability. Eur J Phys Rehabil Med.

[12] STROBE. STROBE Statement http://www.strobe-statement.org/?id=available-checklists2007 [

[13] Kelln BM, McKeon PO, Gontkof LM, Hertel J (2008). Hand-held dynamometry: reliability of lower extremity muscle testing in healthy, physically active,young adults. J Sport Rehabil.

[14] Hiller CE, Refshauge KM, Herbert RD, Kilbreath SL (2007). Balance and recovery from a perturbation are impaired in people with functional ankle instability. Clin J Sport Med.

[15] Augustsson SR, Nae J, Karlsson M, Peterson T, Wollmer P, Ageberg E (2021). Postural orientation, what to expect in youth athletes? A cohort study on data from the Malmo Youth Sport Study. BMC Sports Sci Med Rehabil.

[16] Gustavsson A, Neeter C, Thomee P, Silbernagel KG, Augustsson J, Thomee R (2006). A test battery for evaluating hop performance in patients with an ACL injury and patients who have undergone ACL reconstruction. Knee Surg Sports Traumatol Arthrosc.

[17] Silbernagel KG, Gustavsson A, Thomee R, Karlsson J (2006). Evaluation of lower leg function in patients with Achilles tendinopathy. Knee Surg Sports Traumatol Arthrosc.

[18] Wenning M, Gehring D, Mauch M, Schmal H, Ritzmann R, Paul J (2020). Functional deficits in chronic mechanical ankle instability. J Orthop Surg Res.

[19] Wisthoff B, Matheny S, Struminger A, Gustavsen G, Glutting J, Swanik C (2019). Ankle strength deficits in a cohort of College athletes with chronic ankle instability. J Sport Rehabil.

[20] Ko J, Rosen AB, Brown CN (2018). Functional performance deficits in adolescent athletes with a history of lateral ankle sprain(s). Phys Ther Sport.

[21] Docherty CL, Arnold BL, Gansneder BM, Hurwitz S, Gieck J (2005). Functional-performance deficits in volunteers with functional ankle instability. J Athl Train.

[22] Dabadghav R (2016). Correlation of ankle eversion to inversion strength ratio and static balance in dominant and non-dominant limbs of basketball players. J Sports Med Phys Fitness.

[23] Lin WH, Liu YF, Hsieh CC, Lee AJ (2009). Ankle eversion to inversion strength ratio and static balance control in the dominant and non-dominant limbs of young adults. J Sci Med Sport.

[24] Smith BI, Docherty CL, Simon J, Klossner J, Schrader J (2012). Ankle strength and force sense after a progressive, 6-week strength-training program in people with functional ankle instability. J Athl Train.

[25] World Medical A (2013). World Medical Association Declaration of Helsinki: ethical principles for medical research involving human subjects. JAMA.

